# Molecular Characterization of *Salmonella* Detected along the Broiler Production Chain in Trinidad and Tobago

**DOI:** 10.3390/microorganisms10030570

**Published:** 2022-03-06

**Authors:** Anisa Sarah Khan, Rian Ewald Pierneef, Narjol Gonzalez-Escalona, Meghan Maguire, Cong Li, Gregory H. Tyson, Sherry Ayers, Karla Georges, Woubit Abebe, Abiodun Adewale Adesiyun

**Affiliations:** 1 School of Veterinary Medicine, Faculty of Medical Sciences, University of the West Indies, St. Augustine 999183, Trinidad and Tobago; anisakhan11@gmail.com (A.S.K.); karla.georges@sta.uwi.edu (K.G.); 2 Agricultural Research Council-Biotechnology Platform, Onderstepoort, 100 Old Soutpan Road, Pretoria 0110, South Africa; pierneefr@arc.agric.za; 3 Division of Microbiology, Office of Regulatory Science, Center for Food Safety and Applied Nutrition, Food and Drug Administration, College Park, MD 20740, USA; narjol.gonzalez-escalona@fda.hhs.gov (N.G.-E.); meghan.maguirethon@fda.hhs.gov (M.M.); 4 Division of Animal and Food Microbiology, Office of Research, Center for Veterinary Medicine, U.S. Food and Drug Administration, Laurel, MD 20708, USA; cong.li@fda.hhs.gov (C.L.); gregory.tyson@fda.hhs.gov (G.H.T.); sherry.ayers@fda.hhs.gov (S.A.); 5 Department of Pathobiology, College of Veterinary Medicine, Tuskegee University, 201 Frederick D Patterson Dr, Tuskegee, AL 36088, USA; wabdela@tuskegee.edu; 6 Department of Production Animal Studies, Faculty of Veterinary Science, Onderstepoort, University of Pretoria, Private Bag X04, Pretoria 0110, South Africa

**Keywords:** broiler production chain, *Salmonella*, molecular characterization, whole-genome sequencing, virulence genes, antimicrobial resistance genes, *blaCTX-M-65*, Trinidad and Tobago

## Abstract

This cross-sectional study determined the serovars, antimicrobial resistance genes, and virulence factors of *Salmonella* isolated from hatcheries, broiler farms, processing plants, and retail outlets in Trinidad and Tobago. *Salmonella* in silico serotyping detected 23 different serovars where Kentucky 20.5% (30/146), Javiana 19.2% (28/146), Infantis 13.7% (20/146), and Albany 8.9% (13/146) were the predominant serovars. There was a 76.0% (111/146) agreement between serotyping results using traditional conventional methods and whole-genome sequencing (WGS) in in silico analysis. In silico identification of antimicrobial resistance genes conferring resistance to aminoglycosides, cephalosporins, peptides, sulfonamides, and antiseptics were detected. Multidrug resistance (MDR) was detected in 6.8% (10/146) of the isolates of which 100% originated from broiler farms. Overall, virulence factors associated with secretion systems and fimbrial adherence determinants accounted for 69.3% (3091/4463), and 29.2% (1302/4463) counts, respectively. Ten of 20 isolates of serovar Infantis (50.0%) showed MDR and contained the *bla_CTX-M-65_* gene. This is the first molecular characterization of *Salmonella* isolates detected along the entire broiler production continuum in the Caribbean region using WGS. The availability of these genomes will help future source tracking during epidemiological investigations associated with *Salmonella* foodborne outbreaks in the region and worldwide.

## 1. Introduction

Since the 1950s, *Salmonella* has been highlighted as an economically important zoonotic pathogen by the World Health Organization (WHO) and the Food and Agriculture Organization of the United Nations (FAO) [1]. The ability of *Salmonella* to cause self-limiting gastroenteritis, coupled with high mortality rates in humans due to invasive infections are causes for public health concerns [2,3]. While many animals serve as reservoirs for *Salmonella*, poultry, and poultry products are one of the primary sources of salmonellosis in humans. Therefore, the possibility of transmission from reservoirs to other animals and humans is concerning. This is compounded by antimicrobial-resistant *Salmonella* strains within the environment, necessitating surveillance and control measures among suspected reservoirs such as chickens. In addition, *S.* Typhimurium and *S.* Enteritidis are of public health significance due to their ability to cause disease in humans and animals in developed and developing countries. However, variations in *Salmonella* serovar distribution have been reported in different countries and are said to be a function of geographic location [4,5].

The use of antimicrobial agents in food-producing animals has been implicated in developing multidrug-resistant (MDR) microorganisms and spreading them through the food chain [6,7]. Of importance to human health, some cephalosporins (β-lactams), quinolones, and aminoglycosides have been classified by the World Health Organization (WHO) as critically important agents since they are used in the treatment of extra-intestinal salmonellosis [8]. The use of ciprofloxacin and ceftiofur as the established therapy protocol for human salmonellosis could be jeopardized as genetic mechanisms promoting MDR isolates have been reported [9]. β-lactamases constitute the primary mechanism of cephalosporin resistance via enzymatic modification, where different genes are implicated. The extended-spectrum β-lactamases (ESβL) include certain alleles of *bla*_TEM_, and all alleles of *bla*_CTX-M_ and *bla_SHV_* genes. Extended-spectrum cephalosporins can also be hydrolyzed by the AmpC β-lactamases, of which *bla*_CMY_ is the most common of particular importance. Quinolone resistance was initially known to develop through chromosomal mutations [10]. However, the recent emergence of plasmid-mediated quinolone resistance (PMQR) mechanisms has been reported. These include *qnr* genes: *qnrA*, *qnrB*, *qnrS*, *qnrC*, and *qnrD*, that encode pentapeptide repeat proteins that bind to and protect DNA topoisomerase IV from inhibition by quinolones, the *aac (6′)-Ib-cr* (modified acetyltransferase) and *qepA* (efflux pump) genes, respectively [11,12]. Plasmids are traditionally known to carry antimicrobial-resistant genes and several virulence-associated traits; however, other resistance mechanisms have been reported in *Salmonella* elsewhere [13,14]. Increasing trends of resistance to quinolones and 3rd generation cephalosporins such as ciprofloxacin and ceftiofur in clinical isolates have led to the introduction of carbapenems and colistin as critical antibiotics of last resort in human salmonellosis [8]. However, the use of colistin to treat both humans and animals has resulted in the emergence of mobilized colistin resistance (*mcr*) genes [15,16]. To date, nine variants of *mcr* genes have been detected in *Salmonella* isolated from humans and animals [16,17,18].

Similar genetic determinants conferring resistance to aminoglycosides, tetracyclines, beta-lactams, and fluoroquinolones have been detected in *Salmonella* strains isolated from livestock and humans, concluding that food and environmental contamination from livestock are carriers of antimicrobial-resistant (AMR) *Salmonella* and are sources of infection to humans [19,20,21]. Thus, it is critical to investigate the resistance profiles and phenotypes they exhibit, and the mutations responsible for resistance using molecular analysis methods.

Therefore, the objectives of this study were to determine the genotypic profiles (serovar, antimicrobial resistance, and virulence factors) of *Salmonella* isolated from various stages of the broiler production–processing–retailing chain in Trinidad and Tobago.

## 2. Materials and Methods

### 2.1. Sample Selection

A total of 146 isolates of *Salmonella* used in this study originated from prior studies conducted at hatcheries and broiler farms [22], broiler processing plants [23], and retail outlets (pluck shops and supermarkets) [24,25]. The type of samples collected from the various studies are as follows, hatcheries: broken eggshells, eggs in the hatcher, eggs in the incubator, hatcher environmental swabs, hatcher fluff, and stillborn chicks; broiler farms: boot swabs, cloacal swabs, litter drag swabs, feed, and water samples—in-house supply and storage tank; processing plants: chilled chicken parts, chilled whole carcasses, neck skins, pre-evisceration carcasses, and post-evisceration carcasses; retail outlets: chicken carcasses. From a total of 207 duplicate isolates (from different enrichment and selective media) of *Salmonella*, which represented 23 serovars from the aforementioned sources, the selected 146 isolates were representatives of the serovars recovered from all *Salmonella*-positive samples. Briefly, samples were processed to isolate *Salmonella* using two enrichments broths, Rappaport-Vassiliadis Soya (RVS) and tetrathionate (TT) (Oxoid, Hampshire, England), and two selective agar, brilliant green agar (BGA) and xylose lysine tergitol 4 (XLT-4) selective media (Oxoid, Hampshire, England) [26]. Suspected *Salmonella* colonies (pink isolated colonies on BGA, red colonies with black centers on XLT-4) were subjected to biochemical tests for identification of *Salmonella* spp. using standard methods [27]. Isolates of *Salmonella* recovered from the four combinations of media (RVS/BGA, RVS/XLT-4, TT/BGA, and TT/XLT-4) were initially screened using the conventional slide agglutination test. Thereafter, 146 non-duplicate isolates of *Salmonella,* randomly selected to represent the serovars and positive samples were subjected to whole-genome sequencing. The following is a summary of the number of isolates included from earlier studies: hatcheries (*n* = 10), farms (*n* = 20), processing plant (*n* = 61), and retail outlets (*n* = 55). Five additional human clinical isolates of *Salmonella* obtained from the Caribbean Public Health Agency (CARPHA) were included in our panel of isolates subjected to WGS.

### 2.2. DNA Extraction and Sequencing

DNA was extracted using the Maxwell RSC cultured cells DNA kit with a Maxwell RSC instrument (Promega Corporation, Madison, WI, USA) following the manufacturer’s protocols for Gram-negative bacteria with additional RNase treatment. DNA concentrations were measured with a Qubit fluorometer (Life Technologies, Carlsbad, CA, USA), standardized to 0.2 ng/µL, and the samples were stored at 4 °C before library preparation.

Whole-genome sequencing (WGS) of the *Salmonella* isolates was performed by the Public Health Agency of Canada (PHAC) Laboratory and Food and Drug Administration (FDA): Center for Food Safety and Applied Nutrition genomics laboratory (FDA-CFSAN) and Center for Veterinary Medicine (FDA-CVM), Maryland, USA. The WGS data was generated on an Illumina MiSeq using 2× 250 bp and 2 × 300 bp paired-end chemistry (Illumina Inc., San Diego, CA, USA) according to the manufacturer’s instructions, at 50–150X coverage. According to the manufacturer’s instructions, the libraries were constructed using 100 ng of genomic DNA using the Illumina DNA Prep (M) Tagmentation kit (Illumina Inc., San Diego, CA, USA) and the Nextera XT kit (Illumina Inc., San Diego, CA, USA).

### 2.3. Genomic Data Analysis and In Silico Determination of Genetic Elements

Quality control including adapter removal of the raw data was conducted using BBDuk (v.37.90; https://jgi.doe.gov/data-and-tools/bbtools/bb-tools-user-guide/bbduk-guide/, accessed on 23 July 2021); sourceforge.net/projects/bbmap/). SPAdes v.3.12.0 [28] was used to create a de novo assembly of each isolate. Only contigs larger than 500 bp were retained for further analysis. Serovar prediction was made using command-line version of SISTR [29] (Version: sistr_cmd v.1.1.1).

Gene finding in each isolate was performed with Prodigal v.2.6.3 [30] (parameters -c -n).

VFDB [31] was used to assign virulence factors. This was carried out with the predicted genes (amino acid format) from Prodigal using NCBI-blast-2.9.0+. Results were filtered for the top hit with 100% identity and 100% alignment length.

CARD [32] was used to assign antimicrobial resistance. This was performed with the predicted genes (amino acid format) from Prodigal using NCBI-blast-2.9.0+. Results were filtered for the top hit with 100% identity and 100% alignment length.

### 2.4. Phenotypic Methods Used for Comparison with WGS

Conventional serotyping methods using the phase reversal technique described previously [22,23,25] were performed at the Public Health Laboratory, Ministry of Health, St. Michael, Barbados. Antimicrobial resistance determined by the disk diffusion method [22,23,24] described previously was also used. Data generated from these two methods were compared to the genomic data.

### 2.5. Statistical Analyzes

R version 4.0.2 was used for Chi-square analysis and data visualization.

### 2.6. Data Deposition

The draft genome sequence of all *S. enterica* strains have been deposited at GenBank under the accession listed in Table S1—Metadata of 146 *Salmonella* isolates detected along the broiler production chain in Trinidad and Tobago.

## 3. Results

### 3.1. Serotyping Results

Overall, the 146 isolates of *Salmonella* subjected to conventional serotyping methods were classified into 23 serovars and 3 unspecific groups (unknown serotype). In silico analysis of the WGS data generated from these 146 isolates using the SISTR software identified 23 different serovars where Kentucky 20.5% (30/146), Javiana 19.2% (28/146), Infantis 13.7% (20/146), and Albany 8.9% (13/146) were the predominant serovars. There was a 76.0% (111/146) agreement in the test results of both methods. Isolates classified as *S.* Albany (*n* = 2), Gaminara (*n* = 2), Oranienburg (*n* = 1), and Soerenga (*n* = 1) by SISTR were all classified as *S.* Infantis (*n* = 6) using the traditional method. Three *S.* Warragul isolates detected using the conventional method were classified as *S.* Caracas on SISTR analysis. The distribution of serovars of *Salmonella* isolates from various sources is shown in Table 1.

### 3.2. Antimicrobial Resistance Profiles

A total of 71 ARO accessions (Antibiotic Resistance Ontology, as defined by CARD) were detected among 22 isolates. Genes associated with aminoglycoside resistance, i.e., *aac(3)-IV* (plasmid-encoded), *aac(6′)-Iaa* (chromosomal- encoded), *aac(6′)Iy* (chromosomal-encoded), *aph(3′)-Ia* (plasmid-encoded), and *aph(4)-Ia* (plasmid-encoded) (Table 2) were found at frequencies ranging from 1.4% to 7.5%. All our *S.* Manhattan and *S.* Aberdeen strains containing the often silent, chromosomal-encoded *aac(6′)-Iaa* and *aac(6′)Iy* genes, exhibited phenotypic aminoglycoside resistance. Ten (6.8%) of 146 isolates contained the *bla_CTX-M-65_* gene, which confers cephalosporin resistance. This gene was identified in *S.* Infantis isolates only. Genes *qacEDelta1* and *sul1*, responsible for antiseptic and sulfonamide resistance, were each detected at a frequency of 8.2% (12/146). *mcr-9*, the mobilized and plasmid-mediated colistin resistance gene, was found in only one isolate. Table 3 shows the distribution of AROs among *Salmonella* isolates from various sources. Isolates from broiler farms accounted for 83.1% (59/71) of AROs where the predominance of *aac(3)-IV* (9.9%; 7/71), *aph(4)-Ia* (9.9%; 7/71), *qacEdelta1* (9.9%; 7/71), *sul1* (9.9%; 7/71), and *bla_CTX-M-65_* (9.9%; 7/71) among cloacal swab isolates (62.7%; 37/59) was evident. *Salmonella* isolated from the water supply at farms (18.6%; 11/59) were found to contain 66.7% (6/9) of the AROs found in this study except for *mcr-9.1*, *aac(6′)-Iaa* and *aac(6′)-Iy*.

Overall, 6.8% (10/146) MDR (resistance to 3 or more classes of antimicrobial agents, according to CARD classification) isolates were detected, of which 100% were recovered at broiler farms and belonged to serovar Infantis.

### 3.3. Virulence Profile

Overall, for the *Salmonella* strains from the four sources (hatcheries, farms, processing plants, and retail outlets), 4463 different virulence factors belonging to five virulence classes were identified. Genes classified as secretion systems and fimbrial adherence determinant classes accounted for the predominant virulence classes of 69.3% (3091/4463) and 29.2% (1302/4463) counts, respectively. Magnesium uptake, stress adaptation, and toxin classes accounted for less than 1.3% (56/4463) counts, respectively. *Salmonella* isolates (*n* = 10) recovered from the hatcheries contained virulence factors belonging to secretion systems (4.2%, 187/4463) and fimbrial adherence determinants (2.0%, 91/4463), whereas farm isolates (*n* = 20) were found to contain fimbrial adherence determinants, 4.4% (198/4463), and secretion system, 10.1% (451/4463). Processing plant *Salmonella* isolates (*n* = 61) contained predominantly factors in the secretion systems, fimbrial adherence determinants, and toxins, accounting for 30.0% (1341/4463), 12.4% (553/4463), and 0.6% (26/4463) count, respectively. Retail outlet isolates (*n* = 55) contained fimbrial adherence determinants, 10.3% (460/4463), secretion system, 24.9% (1112/4463), and toxin-related factors, 0.6% (27/4463). The differences in the detection of virulence factors among the sources were statistically significant (*p* < 0.001).

Serovars Kentucky, Javiana, and Infantis contained higher numbers of virulence factors (all related to secretion systems), accounting for 13.0% (578/4463), 12.1% (540/4463), and 12.1% (517/4463), respectively, of the virulence factors (Table S2). Therefore, it is pertinent to mention that they were the predominant serovars detected in this study.

*S.* Infantis isolates contained factors associated with secretion systems (TTSS-1 translocated effectors, TTSS-SPI-1-, and TTSS-SPI-2-encoded genes), 12.1% (540/4463), and factors associated with fimbrial adherence determinants (*bcfA*, *D*, *F*, *csg A*, *B*, *C*, *E*, *F*, *G, and lpfB*, *E*), 4.9% (220/4463). For the isolates of *S.* Javiana, 11.6% (517/4463), 4.4% (196/4463), and 0.6% (28/4463) were positive for factors associated with secretion systems, fimbrial adherence determinants (*bcfA*, *csgA*, *C*, *D*, *F*, *G,* and *fimF*), and toxins (*cdtB*), respectively. Only secretion system and fimbrial adherence determinant factors were detected among Kentucky isolates, accounting for 13.0% (578/4463) and 5.9% (263/4463), respectively. *S.* Schwarzengrund, Senftenberg, and Caracas contained predominantly factors associated with secretion systems at frequencies ranging from 1.3% to 2.8%. Seven serovars (Caracas, Chester, Enteritidis, Gaminara, Javiana, Montevideo, and Schwarzengrund) contained virulence factors related to toxins, where the *cdtB* was detected in all except serovar Enteritidis, where the *spvB* gene was detected.

### 3.4. Comparison of Frequency of Detection of Resistance and Virulence Factors in Salmonella Strains

**Figure 1:**
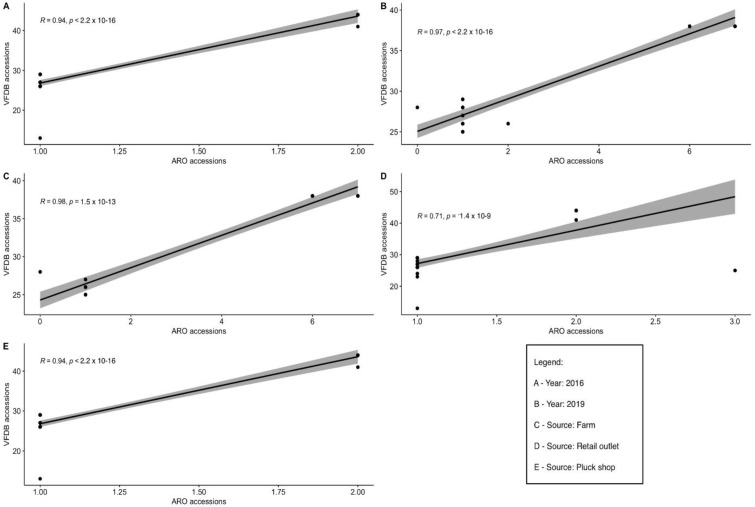
VFDB accessions (virulence) versus ARO accessions (AMR) (**A**–**E**).

Comparisons between the possession of virulence factors (VFDB accessions) and AMR genes (ARO accessions) across serovars were performed and detected 10 sources and years (farm-to-fork, hatcheries, processing plants, retail outlets, ‘pluck shops’, supermarkets, 2016, 2017, 2018, and 2019). Statistically significant positive correlations in *Salmonella* serovars isolated from farms, retail outlets, and ‘pluck shops’, as well as those isolated in 2016 and 2019 were detected, respectively (*p* < 0.05) (Figure 1). Negative and non-significant positive correlations are not displayed.

### 3.5. Detection of ESβL Resistance Genes and Virulence Genes in Isolates of S. Infantis

A comparison of the phenotypic and genotypic resistance patterns in *S.* Infantis isolates is displayed in Table 4. The *bla_CTX-M-65_* gene was only detected among the *S.* Infantis isolates. Of the 10 isolates of serovar Infantis positive for *bla_CTX-M-65_* gene, phenotypically (using the disk diffusion method), two were resistant to two classes of antimicrobial agents, and six were MDR. However, genotypically, all 10 Infantis isolates exhibited MDR. Furthermore, the resistance gene *qacEDelta1* responsible for antiseptic resistance was found in all the 10 serovar Infantis isolates. Additionally, virulence factors associated with fimbrial adherence determinants and the secretion system were detected in all the 10 isolates of serovar Infantis.

## 4. Discussion

This is the first documented WGS study conducted in the poultry (broilers and layers) industry along the broiler production chain in Trinidad and Tobago, and the Caribbean region at large. Whole-genome sequencing analysis has been used to investigate genetic characteristics and phylogenies among *Salmonella* strains isolated from different origins, such as humans, food, animals, and the environment [33,34,35,36]. The current study was comprised of isolates from four cross-sectional studies conducted at the level of retail outlets (2016–2017) [25], broiler processing plants (2018) [23], broiler farms, and broiler hatcheries (2019) [22]. Although several limitations exist with the use of cross-sectional instead of longitudinal studies, this approach provides valuable information on the status of *Salmonella* shedding and contamination at the four levels (hatcheries, farms, processing plants, and retail outlets) of the broiler production chain in the country. Furthermore, the information obtained will lead to a better understanding of the epidemiology of *Salmonella* and the associated public health implications. Finally, this approach will also facilitate the implementation of an effective surveillance system across the poultry production system in the country.

Using the SISTR pipeline, a 76% agreement was detected with the traditional conventional serotyping method, which utilizes the White–Kauffman–Le Minor (WKL) scheme, which is based on immunological reactions to somatic (O) and flagellar (H) antigens [37]. However, it has been documented that conventional serotyping is time-consuming, labor-intensive, costly, and some isolates do not express serotype antigens due to a single nucleotide change in the genome [38,39]. On the other hand, the SISTR pipeline has been validated and a 94.6% overall serovar prediction accuracy was reported when 4291 genomes were analyzed [29]. In silico serotyping channels such as SISTR provide us with an understanding of the antigenic genes carried by an isolate and not necessarily what is expressed by that isolate, an advantage over traditional serotyping methods. In a study that compared three in silico pipelines, SISTR, SeqSero, and MLST to traditional serotyping techniques using a set of 813 verified clinical and laboratory isolates, 94.8%, 88.2%, and 88.3% accuracy, respectively was reported [40]. Of significance in our study was the incorrect serotype classification by the conventional method of potential public health important serotypes such as *S.* Albany, Senftenberg, Infantis, and Caracas. Variations in *Salmonella* serovars in poultry have been reported in different countries and are known to be a function of geographic location [5]. In Egypt [41], serovars Enteritidis and Typhimurium were isolated from broiler chickens at retail outlets; in Japan [42], serovars Infantis, Manhattan, Schwarzengrund from cecal samples in broilers; in China [43], serovars Pullorum, Gallinarum, Enteritidis, and Typhimurium were the predominant serotypes. Unlike our study where *S.* Kentucky, Javiana, Infantis, and Albany were the predominant serovars isolated along the broiler production chain.

Gene *bla*_CTX-M-65_ was detected in 77% (31/40) of the *S.* Infantis strains isolated at Colombian processing plants [44], a finding comparable to the 50% (10/20) detected in the current study. Worldwide, the rapid development of resistance to extended-spectrum cephalosporins, predominantly associated with the production of β-lactamase-producing bacteria (EsβL) in different *Salmonella* serovars, has been reported. In agreement with our study, EsβL resistance genes have been detected in *Salmonella* strains isolated from animal products in several countries, including Korea (food animals and humans), 1.6% [45], Mexico (humans and animals), 6.6% [46], and Brazil (broiler chickens), where 27.8% [47] of isolates were positive for the *bla_CTX-M_* gene. The detection of 6.8% MDR strains among serovar Infantis isolates possessing the *bla_CTX-M-65_* gene is of public health significance due to the reported cross-transmission of EsβL-producing bacterial strains from poultry farms to other livestock farms and humans with the potential for wide-spread population infections [48,49]. A cause for concern is the detection of 10 MDR serovar Infantis strains, each possessing the *bla_CTX-M-65_* gene, in addition to 38 virulence homologs according to VFDB. In 2014, the detection of an MDR emergent *Salmonella* Infantis (ESI) strain, often containing the *bla_CTX-M-65_* gene, was first reported in Israel, and subsequently detected in Italy, Japan, and Russia [50,51,52,53]. However, retrospective sequencing tracked the origins of this clone to South America [54,55]. This ESI strain was documented to carry a large plasmid ESI (pESI) with several antimicrobial resistance, metal, and virulence genes. This clone was detected in retail meats in Tennessee, USA, in 2014, but by 2019 had spread throughout the USA to comprise 29% *Salmonella* isolated from retail chickens and 7% from retail turkey [56]. This clone also accounted for nearly 10% of all human Infantis cases by 2017 in the United States and was highly related to chicken sources [57]. The most frequently described ESβL genotype in Colombia between 1997–2018 was *CTX-M*, which was detected primarily in *S.* Typhimurium (40%; 65/164) and *S.* Infantis (29%; 48/164). Detection of *bla_CTX_* genes has been reported in Latin American countries, such as Brazil and Argentina [58]. The assumption was that cephalosporin resistance development was due to the injection of ceftiofur into fertile eggs at hatcheries to prevent *E. coli*-induced omphalitis in day-old chicks [59]. This assumption was supported by a Canadian study that revealed a strong correlation between this practice and the increase in ceftiofur-resistant strains of *S.* Heidelberg [60]. This practice was not evident at hatcheries in our study, nor were *bla* genes detected among hatchery isolates.

A quick look into the NCBI Pathogen detection browser (https://www.ncbi.nlm.nih.gov/pathogens) allows us to determine that the eight strains of *S.* Infantis (ST32) detected in this study were highly related to the MDR emergent *S.* Infantis strains carrying *bla_CTX-M-65_* (https://www.ncbi.nlm.nih.gov/pathogens/tree#Salmonella/PDG000000002.2405/PDS000089910.160?term=CFSAN103822,%20CFSAN103806,%20CFSAN103805,%20CFSAN103797,%20CFSAN103801,%20CFSAN103796,%20CFSAN103798,%20CFSAN103802, accessed on 2 March 2022) reported in previous studies [50,51,52,53,54,55,56,57]. This highlights the usefulness of WGS approaches for AMR surveillance in a country or region, in this case, Trinidad and Tobago, considering the significant public health and clinical implications resulting from the presence of this large plasmid ESI. The plasmid detected in our *S.* Infantis carrying the CTX-M-65 gene (Accession: CP066336.1) contained 312,952 bp, differing from the plasmids reported in the USA [61] and Italy [52], which ranged from 316,160 to 323,122bp. These eight strains exhibited two resistance profiles: *aac(3)-IVa*, *aadA1*, *aph(3’)-Ia*, *aph(4)-Ia*, *bla_CTX-M-65_*, *dfrA14*, *gyrA_D87Y*, *mdsA, mdsB*, *sul1*, *tet(A)* (4 strains) and *aac(3)-IVa*, *aadA1*, *aph(4)-Ia*, *bla_CTX-M-65_*, *dfrA14*, *gyrA_D87Y*, *mdsA*, *mdsB*, *sul1*, *tet(A)* (4 strains), according to the NCBI database (AMRFinderPlus). Our findings were similar to the *aph(4)-Ia, aac(3)-IVa, aph(3′)-Ic, bla_CTX-M-65_, fosA3*, *floR*, *dfrA14*, *gyrA_D87Y*, *sul1*, *tetA*, *aadA1* pattern detected in the USA [61] and *aph(4)-Ia*, *aac(3)-IVa, aph(3′)-Ic, bla_CTX-M-65_, fosA3, floR, dfrA14, sul1, tetA, aadA1* detected clinically in Italy [52], where both studies used ResFinder.

It must be highlighted that EsβL-producing *K. pneumoniae* was detected in 78.8% (41/52) of clinical isolates originating from a tertiary care hospital in Trinidad and Tobago, where the *bla_SHV_* and *bla_CTX-M_* genes were predominantly detected [62]. It is of interest that all the MDR Infantis strains isolated in the current study originated from broiler farms. This is because there is a potential for *Salmonella* strains positive for *bla_CTX_* gene, AMR, and associated virulence genes, to enter the human food chain through the processing plants and chicken products at the retail outlets. This is supported by reports documenting close association of MDR Infantis strains recovered from the broiler population to animal production environments, eventually spreading into the food chain and potentially humans [63,64].

As with this study, aminoglycoside resistance genes and *sul1* genes were also detected in *Salmonella* Infantis strains isolated in a recent study conducted at three Colombian broiler processing plants [44]. Sulphonamide resistance conferred by *sul* genes [65] was reported in Canadian swine and chicken *Salmonella* isolates [66] and at a broiler processing plant in China [67]. However, in our study, only the *sul1* gene was detected in all our *S.* Infantis strains and the only Senftenberg strain assessed. Arkali et al. [68] detected the *sul1* gene among 58% of *Salmonella* isolated from chickens in Eastern Turkey. The detection of one mobilized colistin resistance *mcr-9.1* gene [69] in an isolate of serovar Senftenberg was not a significant finding. This gene is not associated with colistin resistance in *Salmonella* or *E. coli* in the United States [70]. However, detecting this novel *mcr-9* homolog is crucial as it can confer phenotypic resistance to colistin and warrants close monitoring [16].

The *qacEdelta1* gene, known to confer resistance to antiseptics, was also detected in *Salmonella* from retail foods of animal origin [20]. It must be considered that the presence of antimicrobial resistance genes can represent the phenotypic resistance of antimicrobial agents, and thus diminish their effectiveness when used on farms or processing plants. However, it is important to mention that there are several mechanisms of antimicrobial resistance in bacteria. It is not always associated with a specific gene responsible for resistance. This supports our findings where resistance genes were found in two Infantis strains, but they were all sensitive phenotypically. Cross-resistance to antimicrobial agents can occur with resistance within group members of chemical-related compounds, and/or with a similar mechanism of action [71,72]. The correlation of genotypic and phenotypic resistance was variable in our study, contrary to the findings of other studies where the harmonic correlation was evident [67,73]. The lack of correlation between phenotypic and genotypic resistance profiles may occur due to the low sensitivity and specificity of the disk method, inoculum concentration, laboratory capacity, and individual skill. Misalignments between phenotypic and genotypic resistance patterns have been reported by others [74,75].

In the current study, only 6.8% (10/146) of the isolates, based on genotypic characterization, exhibited multidrug resistance, at variance with the 96.6% reported in *Salmonella* isolated from chickens sampled at chicken farms in South Africa [76] and the 27.3% reported for *Salmonella* strains isolated from broilers in Egypt [77]. Therefore, our low frequency of detecting MDR is of therapeutic significance at the broiler farm level in the country.

*SPI-1* and *SPI-2* genes enable the invasion of eukaryotic cells, induction of macrophage cytotoxicity, invasion of phagocytes, and survival inside phagocytic cells [78,79,80,81]. The inactivation of the TTSS-1 translocated effector gene *sipB in S.* Dublin has been associated with reduced fluid secretion and inflammation [82]. This is of public health significance because of the 73 genes detected in the current study, 49.3% and 35.6% were detected in *Salmonella* strains isolated from processing plants and retail outlets, respectively, highlighting the risk posed to consumers should they be infected with a serovar positive for the gene. In the current study, serovars Aberdeen, Anatum, Enteritidis, Infantis, Javiana, Manhattan, Virchow, and Weltevreden were all positive for the *sipB* gene.

Fimbrial adherence factors that aid intestinal adhesion such as long polar fimbriae (*lpfA*) and aggregative fimbriae (*agfA/csgA*) are highly conserved in *Salmonella* and have been implicated in biofilm formation and adhesion to surfaces and epithelial cells that is an important stage before biofilm formation, respectively [83,84]. This is important in the current study because 99.3% of the isolates were positive for the *csgA* gene, therefore having the potential for biofilm formation and persistence in the environment. In addition, the high incidence of *csgA* in our study is comparable to the findings in different serovars, as reported by others [85,86].

Typhoid toxin/*cdtB* cytolethal distending toxin B, previously thought to be a unique virulence factor in *S.* Typhi, was recently characterized in at least 40 non-typhoidal *Salmonella* serovars [87], as evident in our study. The detection of virulence genes *invA*, *csgA*, *lpfA*, *sopE*, and *spvC* in our *S.* Enteritidis strains agrees with the findings of studies conducted on chickens sold at Bangladeshi retail outlets [88], in food and humans in Brazil [89], and in humans and animals in Iran [90].

The positive correlations in the detection of AMR and virulence genes in the *Salmonella* serovars isolated from farms, retail outlets, and ‘pluck shops’ are indicative of close similarities in the occurrence of AMR and virulence genes in different serovars and isolates in the study area or source-dependent AMR/virulence profiles. The presence of virulence genes and the occurrence of AMR *Salmonella* isolates can potentially accelerate the pathogenicity of microbes [91]. It has also been reported that the emergence of resistant *Salmonella enterica* solely depends on genetic and pathogenicity mechanisms that may enhance survivability by preserving their drug resistance genes [92]. However, the correlation between AMR and virulence has been shown to vary in studies conducted by others. The acquisition of AMR by *Salmonella* isolates decreases [93,94], increases [95,96], or does not change [97,98] their potential virulence according to those authors.

## 5. Conclusions

This study highlighted the antimicrobial resistance and virulence genes associated with *Salmonella* serovars isolated along the broiler production chain in Trinidad and Tobago. The detection of the *bla_CTX-M-65_* gene, MDR, and highly virulent *S.* Infantis isolates based on their genotypes, is cause for concern given their international emergence and implications for human health. The positive correlation of resistance and virulence genes detected at broiler farms, processing plants, and retail outlets (‘pluck shops’) is significant since the latter two stages of the broiler continuum can directly impact consumers of contaminated, improperly handled, or cooked chicken.

The availability of these genomes will help future source tracking during epidemiological investigations associated with *Salmonella* foodborne outbreaks in the region and worldwide. Therefore, the abundance of data from several sources in the country will benefit the scientific community at large.

## Figures and Tables

**Table 1:** The distribution of serovars of *Salmonella* isolates from various sources based on in silico analysis.

	No. of Strains of *Salmonella* Detected from the Following:				
Serovars	Hatchery	Farm	Processing Plant	Pluck Shop ^a^	Supermarket ^a^
Aberdeen	0	0	1	1	0
Alachua	0	0	1	0	0
Albany	0	4	8	1	0
Anatomy	0	0	5	0	0
Caracas	0	0	0	3	0
Chester	0	0	0	0	2
Enteritidis	0	0	9	0	0
Fresno	1	0	0	0	0
Gaminara	0	3	0	0	0
Infantis	0	11	9	0	0
Javiana	0	0	10	17	1
Kentucky	8	0	7	12	3
Liverpool	0	0	1	0	0
Manhattan	0	0	0	7	0
Mbandaka	0	0	1	0	0
Molade	0	0	0	0	1
Montevideo	0	0	0	2	1
Oranienburg	0	1	0	0	0
Schwarzengrund	0	0	7	1	0
Senftenberg	1	0	0	2	1
Soerenga	0	1	0	0	0
Virchow	0	0	1	0	0
Weltevreden	0	0	1	0	0
Sub-total	10	20	61	46	9

^a^ Retail outlets comprised pluck shops and supermarkets.

**Table 2:** Antimicrobial class and genes detected in 146 *Salmonella* isolates were used in this study.

	Antimicrobial Class and Genes Detected ^a^						
Pattern	Aminoglycoside	Disinfectant	Cephalosporin	Peptide	Sulphonamide	Number of Isolates (%)	Serovar (*n*, %)
Pattern 1	*aph(4)-Ia*	*qacEDelta1*	*bla_CTX-M-65_*	-	*sul1*	6 (4.2)	Infantis (6, 100.0)
	*aac(3)-IV*						
Pattern 2	*aph(3′)-Ia*	*qacEDelta1*	-	-	*sul1*	1 (0.7)	Infantis (1, 100.0)
	*aph(4)-Ia*						
	*aac(3)-IV*						
Pattern 3	*aph(3′)Ia*	*qacEDelta1*	*bla*_CTX-M-65_	-	*sul1*	4 (2.8)	Infantis (4, 100.0)
	*aph(4)-Ia*						
	*aac(3)-IV*						
Pattern 4	-	*qacEDelta1*	-	-	*sul1*	1 (0.7)	Senftenberg (1, 100.0)
Pattern 5	*aac(6′)-Iaa*	-	-	-	-	7 (4.9)	Manhattan (7, 100.0)
Pattern 6	*aac(6′)-Iy*	-	-	-	-	2 (1.4)	Aberdeen (2, 100.0)
Pattern 7	-	-	-	*mcr-9.1*	-	1 (0.7)	Senftenberg (1, 100.0)
Total	20 (14.9)	12 (9.0)	10 (7.5)	1 (0.7)	12 (9.0)	22 (16.1)	

^a^ Of a total of 146 isolates subjected to CARD analyzes, AMR genes were detected in 22 isolates shown, 121 isolates possessing the core gene *golS* (regulator of a multidrug efflux pump) were not included in the table and three isolates were negative for resistance genes (Liverpool, Mbandaka, and Oranienburg).

**Table 3:** Frequency of ARO accessions detected in this study.

			Distribution of AROs among the Various Sampling Levels			
ARO Name ^a^	No. of AROs	Overall Frequency (%) ^b^	Hatchery	Farm	Processing Plant	Retail Outlet
*aac(3)-IV*	11	7.5	0	11	0	0
*aac(6‘)-Iaa*	7	4.8	0	0	0	7
*aac(6‘)-Iy*	2	1.4	0	0	1	1
*aph(3‘)-Ia*	5	3.4	0	5	0	0
*aph(4)-Ia*	11	7.5	0	11	0	0
*bla_CTX-M-65_*	10	6.8	0	10	0	0
*mcr-9.1*	1	0.7	1	0	0	0
*qacEDelta1*	12	8.2	0	11	0	1
*sul1*	12	8.2	0	11	0	1
*Total*	71		1	59	1	10

^a^ Antibiotic-resistant ontology name in accordance with the Comprehensive Antibiotic Resistance Database (CARD) software. ^b^ A total of 71 ARO counts were detected in 146 isolates.

**Table 4:** Detection of the *bla_CTX-M-65_* gene, other resistance genes, and virulence genes in *S.* Infantis.

BioSample	Isolate No. ^a^	Phenotypic AMR Using the Disk Diffusion Method ^b,c,d^							Genotypic Characteristics ^e^	
		P	TE	CE	AM	PH	S	F	Other Resistance Genes Detected ^f^	Virulence Factors
SAMN25867756	F 17	S	R	R	R	S	R	S	*qacEDelta1*	*agf/csg*
									*aph(4)-Ia*	*bcf*
									*aac(3)-IV*	*lpf*
									*sul1*	TTSS (SPI-1 encode)
										TTSS (SPI-2 encode)
										TTSS-1 translocated effectors
SAMN25867757	F 22	S	R	R	R	S	R	S	*qacEDelta1*	*agf/csg*
									*aph(4)-Ia*	*bcf*
									*aac(3)-IV*	*lpf*
									*sul1*	TTSS (SPI-1 encode)
										TTSS (SPI-2 encode)
										TTSS-1 translocated effectors
SAMN14677229	F 11	S	R	R	R	S	R	S	*qacEDelta1*	*agf/csg*
									*aph(4)-Ia*	*bcf*
									*aac(3)-IV*	*lpf*
									*sul1*	TTSS (SPI-1 encode)
										TTSS (SPI-2 encode)
										TTSS-1 translocated effectors
SAMN14677211	F 32	S	R	R	R	S	R	S	*aph(3′)-Ia*	*agf/csg*
									*qacEDelta1*	*bcf*
									*aph(4)-Ia*	*lpf*
									*aac(3)-IV*	TTSS (SPI-1 encode)
									*sul1*	TTSS (SPI-2 encode)
										TTSS-1 translocated effectors
SAMN14677232	F 36	S	R	R	R	S	R	S	*aph(3′)-Ia*	*agf/csg*
									*qacEDelta1*	*bcf*
									*aph(4)-Ia*	*lpf*
									*aac(3)-IV*	TTSS (SPI-1 encode)
									*sul1*	TTSS (SPI-2 encode)
										TTSS-1 translocated effectors
SAMN14677210	F 2	S	S	S	S	S	S	S	*qacEDelta1*	*agf/csg*
									*aph(4)-Ia*	*bcf*
									*aac(3)-IV*	*lpf*
									*sul1*	TTSS (SPI-1 encode)
										TTSS (SPI-2 encode)
										TTSS-1 translocated effectors
SAMN14677203	F 4	S	R	R	R	S	S	R	*qacEDelta1*	*agf/csg*
									*aph(4)-Ia*	*bcf*
									*aac(3)-IV*	*lpf*
									*sul1*	TTSS (SPI-1 encode)
										TTSS (SPI-2 encode)
										TTSS-1 translocated effectors
SAMN14677209	UWI-F30	S	S	S	S	S	S	S	*aph(3′)-Ia*	*agf/csg*
									*qacEDelta1*	*bcf*
									*aph(4)-Ia*	*lpf*
									*aac(3)-IV*	TTSS (SPI-1 encode)
									*sul1*	TTSS (SPI-2 encode)
										TTSS-1 translocated effectors
SAMN14677207	UWI-F9	S	R	S	R	S	S	S	*qacEDelta1*	*agf/csg*
									*aph(4)-Ia*	*bcf*
									*aac(3)-IV*	*lpf*
									*sul1*	TTSS (SPI-1 encode)
										TTSS (SPI-2 encode)
										TTSS-1 translocated effectors
SAMN14677208	UWI-F31	S	R	S	R	S	S	S	*aph(3′)-Ia*	*agf/csg*
									*qacEDelta1*	*bcf*
									*aph(4)-Ia*	*lpf*
									*aac(3)-IV*	TTSS (SPI-1 encode)
									*sul1*	TTSS (SPI-2 encode)
										TTSS-1 translocated effectors

^a^ All 10 isolates were obtained from broiler farms comprising 7 (70%) cloacal swabs, 2 (20%) water supply (UWI-F30 and UWI-F9), and 1 drag swab of litter (UWI-F31) from where *blaCTX-M-65*, the only *ESβL*-resistance gene was detected. ^b^ P, penam (amoxicillin–clavulanic acid, 30 µg); TE, tetracycline (doxycycline, 30 µg); CE, cephalosporin (ceftriaxone, 30 µg); AM, aminoglycoside (gentamicin, 10 µg, and kanamycin, 30 µg); PH, phenicol (chloramphenicol, 30 µg); S, sulphonamide (sulfamethoxazole–trimethoprim, 23.75 and 1.25 µg); F, fluoroquinolone (ciprofloxacin, 5 µg). ^c^ A total of 146 (151 with controls) isolates of *Salmonella* were tested for AMR by the disk diffusion method, resistance genes, and virulence genes by WGS where 6.6% (10/151) were positive for *ESβL* resistance genes (*bla_CTX-M-65_*). ^d^ S: Susceptible and R: Resistance. ^e^ Antimicrobial resistance and virulence analyses were performed using CARD and VFDB. ^f^ All isolates belonged to serovar Infantis and contained the *golS* gene, not shown.

## Data Availability

All the data are contained within the article and the Supplementary Materials.

## Supplementary Materials

The following are available online at https://www.mdpi.com/article/10.3390/microorganisms10030570/s1, Table S1: Metadata of 146 *Salmonella* isolates detected along the broiler production chain in Trinidad and Tobago. Table S2: Distribution of virulence genes and classes among the various serovars of *Salmonella* isolated in this study.
